# PreZon: Prediction by Zone and Its Application to Egg Productivity in Chickens

**DOI:** 10.1100/2012/785187

**Published:** 2012-05-22

**Authors:** Yen-Jen Lin, Ming Li Liou, Wen Chuan Lee, Chuan Yi Tang

**Affiliations:** ^1^Department of Computer Science, National Tsing Hua University, Hsinchu City 30013, Taiwan; ^2^Department of Medical Laboratory Science and Biotechnology, Yuanpei University, Hsin-chu City 30015, Taiwan; ^3^Division of Biotechnology, Animal Technology Institute Taiwan, Miaoli 35053, Taiwan; ^4^Department of Computer Science & Information Engineering, Providence University, Taichung City 43301, Taiwan

## Abstract

Taiwan red-feathered country chickens (TRFCCs) are one of the main meat resources in Taiwan. Due to the lack of any systematic breeding programs to improve egg productivity, the egg production rate of this breed has gradually decreased. The prediction by zone (PreZone) program was developed to select the chickens with low egg productivity so as to improve the egg productivity of TRFCCs before they reach maturity. Three groups (*A*, *B*, and *C*) of chickens were used in this study. Two approaches were used to identify chickens with low egg productivity. The first approach used predictions based on a single dataset, and the second approach used predictions based on the union of two datasets. The levels of four serum proteins, including apolipoprotein A-I, vitellogenin, X protein (an IGF-I-like protein), and apo VLDL-II, were measured in chickens that were 8, 14, 22, or 24 weeks old. Total egg numbers were recorded for each individual bird during the egg production period. PreZone analysis was performed using the four serum protein levels as selection parameters, and the results were compared to those obtained using a first-order multiple linear regression method with the same parameters. The PreZone program provides another prediction method that can be used to validate datasets with a low correlation between response and predictors. It can be used to find low and improve egg productivity in TRFCCs by selecting the best chickens before they reach maturity.

## 1. Introduction

Egg production is the main economic trait for laying hens. To improve egg production, systematic breeding programs for the long-term selection of chickens have been used to improve egg production for many years in Western countries [1]. Several selection indices, including body weight, age at onset of laying, rate of egg production, egg size, inter- and intraclutches, and hierarchical follicles, have been used to improve many traits of poultry [2–4]. However, phenotypic measurements of chicken egg characteristics and production traits using those related parameters are usually restricted to mature females. As the chicken genome project nears completion, the number of genes identified is growing rapidly [5]. Marker-assisted selection of immature chickens using quantitative trait loci (QTL), genotyping and gene polymorphisms is a potential approach to accelerate the genetic improvement of these traits in the chicken population [6–8]. Thus far, these genetic approaches have typically been restricted to long-term bred populations rather than randomly bred populations.

The traditional notion for marker-assisted selection within a chicken population is primarily based on phenotypic traits that are associated with egg production [9, 10]. These phenotypic measurements of production traits have typically been restricted to laying hens. Recently, selection indices incorporating phenotypic and genotypic traits have been investigated [6]. Several selection approaches, including phenotype, selection index, and best linear unbiased prediction (BLUP), have been used to estimate breeding values [11, 12]. One computational model of mating strategy in a controlled breeding program provides a novel viable and robust approach to designing [13]. Thus far, these selection programs have been restricted to inbreeding or to a closed line.

Taiwan red-feathered country chickens (TRFCCs) have become very popular in Taiwan because of their meat performance. TRFCCs originated in north and south Asia and have been crossed for many years. Owing to the lack of any systematic breeding program to improve egg productivity, the breeding cost has been increasing in Taiwan, while the breeding efficiency has decreased [14]. Based on their strong tendency for broodiness, an egg production of 120–150 per hen per year has proposed the limit for reproduction [15]. Nowadays improving egg production has become an important issue for stimulating market competitiveness in Taiwan.

To improve egg production, the selection of chickens for increased egg number or laying rate using proteomic approaches has become a possible alternative. Huang et al. [16] investigated serum protein profiles during the development of chickens and found that the levels of 13 proteins differed during developmental stages. Kuo et al. [9] analyzed the expression levels of hypothalamic proteins between high and low egg-producing strains of chickens and found differences in expression levels between both groups, revealing that protein levels may serve as molecular markers to select for egg productivity. Leszczynski et al. [10] estimated egg production by evaluating plasma levels of estradiol and progesterone. Our previous study showed that serum protein levels are associated with egg production at the peak egg production stage [17]. The results of these studies imply that protein levels may serve as valuable parameters to improve egg production. How to use such a selection marker to achieve genetic progress remains to be determined.

The prediction by zone provides an excellent model for finding the low egg productively and improving egg productivity by selecting the best chickens before they reach maturity. As the prediction by zone has been patented (Patent no.: US 7,806,079 B2), our study provides a new model in addition to traditional approaches to improve egg productivity.

## 2. Methods and Materials

### 2.1. Description of the Problem

As the variables are not associated with validation variable, *E*, the prediction of the unknown dataset using the known dataset using traditional statistical methods would not be successful. For example, we are given two sets *A* and *B* of multivariate data with *n*_*A*_ and *n*_*B*_ objects, respectively, each of which contains the scores for *m* variables, *x* ^1^, *x* ^2^,…, *x* ^*p*^,…, *x* ^*m*^, where  1 ≤ *p* ≤ *m*, and the known dataset has a validation variable *E*. The values for objects *i* = 1, 2,…, *n*_*A*_ in the unknown dataset *A* can then be denoted by *AX*_*i*_ = {*Ax*_*i*_^1^, *Ax*_*i*_^2^,…, *Ax*_*i*_^*m*^}, and the values for object *j* = 1, 2,…, *n*_*B*_ in the known dataset *B* can then be denoted by *BX*_*j*_ = {*Bx*_*i*_^1^, *Bx*_*j*_^2^, ..., *Bx*_*j*_^*m*^}. However, the variable *j* in the known dataset *B* is the identity variable. The variables are not strongly associated with the validation variable *E* according to Pearson's correlation coefficient. The prediction by zone is used to predict the subset validation variable in *AX* using the known dataset *BX* and *E*. One example is to select chickens with low egg productivity in the three batches of TRFCCs using this algorithm. The levels of four serum proteins, apolipoprotein A-I, vitellogenin, X protein (an IGF-I-like protein), and apo VLDL-II for the three batches of TRFCCs were measured at the indicated ages. The selection approach, termed zone, was performed at the indicated time period using serum protein levels as selection parameters. The selection values were then estimated and compared to those of the first-order multiple linear regression method.

### 2.2. Algorithm

In this section, the PreZone is described. In the first subsection, we present the preprocessing for obtaining the transferred score table and the transferred rank table for the unknown dataset and for obtaining the score table and rank table in the known dataset. Following the subsection, the zone table is generated by Algorithms 1 and 2. In the last subsection, we use the candidate zone to obtain the predicted variables.

#### 2.2.1. Preprocessing

**Algorithm 1**

(1) Rank the score variables for the unknown dataset *A*, *Ax*_*i*_^*p*^ to obtain the rank variables, *As*_*i*_^*p*^, where *i* = 1,…, *n*_*A*_ and *p* = 1,…, *m*.

(2) Rank the score variables for the known dataset, *Bx*_*j*_^*p*^ to obtain the rank variables, *Bs*_*j*_^*p*^, where *j* = 1,…, *n*_*B*_ and *p* = 1,…, *m*.

(3) Rank the validation variables, *E*, and then choose the lower subset as the validation candidate dataset.

(4) From the validation candidate dataset to order the *E*_*j*_ to get the order *ej* = 1,2,…, *cn*_*B*_, then *Bx*_*ej*_^*p*^  and *Bs*_*ej*_^*p*^, and these values denote the candidate score and candidate rank, respectively.

The unknown dataset *A* was ranked by the score variables, and *As*_*i*_^*p*^ is the rank variable. The same process that was just applied to the known dataset *B* was used. But then the rank variable was generated, *Bs*_*j*_^*p*^.  Table 1 shows the rank and score variables for the unknown set *A* and known set *B*. For the known dataset *B*, according to the validation variable, *E*, choose the lower validation objects. We order the *E*_*j*_  values to obtain the order *ej* = 1,2,…, *cn*_*B*_, and we generated the candidate score variables, *Bx*_*ej*_^*p*^, and candidate rank variables *Bs*_*ej*_^*p*^ in Table 2.

#### 2.2.2. Zone Algorithm

Given *Ax*_*i*_^*p*^, *As*_*i*_^*p*^, *Bx*_*ej*_^*p*^  and *Bs*_*ej*_^*p*^, where *p* = 1,…, *m* and *ej* = 1,2, …, *cn*_*B*_, the following algorithm was used to generate the zone. We used *Bc*_*ej*_^*p*^ and *Ac*_*i*_^*p*^ to denote the validation candidate dataset zone and the unknown dataset zone, respectively.

**Algorithm 2**

(1) In the unknown dataset, the transferred score *Ax* *t*_*i*_^*p*^ and the transferred rank score *As* *t*_*i*_^*p*^ were generated by the following:

(1) $$\begin{array}{cc} Ast_{i}^{p} & =As_{i}^{p}\times \frac{n_{B}}{n_{A}},\\ Axt_{i}^{p} & =\left(Ax_{i}^{p}-\text{mean}\left(Ax_{•}^{p}\right)\right)\times \frac{\text{S}.\text{D}.\left(Bx_{•}^{p}\right)}{\text{S}.\text{D}.\left(Ax_{•}^{p}\right)}\\ & \;+\text{mean}\left(Bx_{•}^{p}\right). \end{array}$$

(2) For every *P*, *Bx*_*ej*_^*p*^ and *Bs*_*ej*_^*p*^ are used to find the zone from the unknown dataset *A*.

(2.1) According to the *As* *t*_*i*_^*p*^ to generate order of the unknown set *A*.

For validation candidate data, first *Bx*_1_^*p*^ and *Bs*_1_^*p*^ are used to find the first zone in the order of the unknown dataset *A*.

(2.1.1) Case I: When  *As* *t*_*pi*_^*p*^ ≤ *Bs*_1_^*p*^ and *As* *t*_*pi*+1_^*p*^ > *Bs*_1_^*p*^, then  *Ac*_*pi*_^*p*^ = 1. If *Ax* *t*_*pi*+1_^*p*^ ≤ *Bx*_1_^*p*^, we keep to add *pi*, until *Ax* *t*_(*pi*+*k*−1)_^*p*^ ≤ *Bx*_1_^*p*^  and *Ax* *t*_(*pi*+*k*)_^*p*^ ≥ *Bx*_1_^*p*^, where *k* is integer number. When the objects are in the [*pi*, *pi* + *k*] regions, we defined the zone of the object as {*Ac*_*pi*_^*p*^ = 1,…, *Ac*_*pi*+*k*_^*p*^ = 1}.
(2.1.2) Case II: When *As* *t*_*pi*_^*p*^ ≤ *Bs*_1_^*p*^ and *As* *t*_*pi*+1_^*p*^ > *Bs*_1_^*p*^, then *Ac*_*pi*_^*p*^ = 1. If *Ax* *t*_*pi*_^*p*^ ≥ *Bx*_1_^*p*^, we keep to find *k*′  until *Ax* *t*_(*pi*−*k*′+1)_^*p*^ ≥ *Bx*_1_^*p*^ and *Ax* *t*_(*pi*−*k*′)_^*p*^ ≤ *Bx*_1_^*p*^ where *k*′ is integer number. When the objects are in the [*pi* − *k*′, *pi* + 1] regions, we defined the zone of the object as  {*Ac*_*pi*−*k*′_^*p*^ = 1,…, *Ac*_*pi*+1_^*p*^ = 1}.

(2.2) For every *ej* = 2,…, *cn*_*B*_, *Bx*_*ej*_^*p*^ and *Bs*_*ej*_^*p*^, first to find the *pi*′ at *As* *t*_*pi*′_^*p*^ ≤ *Bs*_*ej*_^*p*^ and  *As* *t*_*pi*′+1_^*p*^ > *Bs*_*ej*_^*p*^. These values must be constrained by one of the following two situations. One is  *Ax* *t*_*pi*′_^*p*^ ≤ *Bx*_*ej*_^*p*^ and *Ax* *t*_*pi*′+*k*^″^_^*p*^ ≥ *Bx*_*ej*_^*p*^ and *Ax* *t*_*pi*′+*k*′′−1_^*p*^ ≤ *Bx*_*ej*_^*p*^ or another is *Ax* *t*_*pi*′_^*p*^ ≥ *Bx*_*ej*_^*p*^ and *Ax* *t*_*pi*′−*k*′′′_^*p*^ ≤ *Bx*_*ej*_^*p*^ and *Ax* *t*_*pi*′−*k*′′′+1_^*p*^ ≥ *Bx*_*ej*_^*p*^  where *k*′′and *k*′′′ are integers. When objects are in the following one of the two regions [*pi*′, *pi*′ + *k*′′] or  [*pi*′ − *k*′′′, *pi*′ + 1], we defined the {*Ac*_*pi*′_^*p*^,…, *Ac*_*pi*′+*k*′′_^*p*^} or {*Ac*_*pi*′−*k*′′′_^*p*^,…, *Ac*_*pi*′+1_^*p*^} as new zone. When the new zone is overlapping previous zone, the zone is the same as previous zone. When the objects are between two zones, these objects generated other zones.

(3) From step (2), we can obtain zone for the unknown dataset *A*. Hence, every object in the unknown dataset *A* has the zone and is denoted as *Ac*_*i*_ = {*Ac*_*i*_^1^, *Ac*_*i*_^2^,…, *Ac*_*i*_^*p*^,…, *Ac*_*i*_^*m*^}.

(4) The each object, *ej*, in the known candidate dataset gets *Bc*_*ej*_ = {*Bc*_*ej*_^1^, *Bc*_*ej*_^2^,…, *Bc*_*ej*_^*p*^,…, *Bc*_*ej*_^*m*^}.

If *n*_*A*_ ≠ *n*_*B*_, the rank in the unknown dataset is transformed into a known dataset by the rank transformation (Table 3(a)). The rule of the transferred rank is *As* *t*_*i*_^*p*^ = *As*_*i*_^*p*^ × *n*_*B*_/*n*_*A*_. When the numbers of elements in the unknown dataset and in the known dataset are different, it is impossible to identify the poultry with the same rank in the two sets, and thus the ranks for unknown dataset *A* need to be transformed. The ranks for the posttransformation unknown dataset *A* that are close to the ranks for the known dataset *B* are selected. The *n*_*B*_/*n*_*A*_ is the ratio of the position in the known dataset. This ratio of the position is also in the unknown dataset, so that it is *As*_*i*_^*p*^ times this ratio. According to (1), the unknown dataset generated a rank similar to that of the known dataset.

Assume that both distributions are normal. The means and standard deviations of the unknown dataset and the known dataset are different. The order transferred score was generated as the following: *Ax* *t*_*i*_^*p*^ = (*Ax*_*i*_^*p*^ − mean(*Ax*_•_^*p*^)) × S.D.(*Bx*_•_^*p*^)/S.D.(*Ax*_•_^*p*^) + mean(*Bx*_•_^*p*^), where mean(*Bx*_•_^*p*^) and S.D.(*Bx*_•_^*p*^) are mean the score and the standard deviation for known dataset *B*, respectively, and mean(*Ax*_•_^*p*^) and S.D.(*Ax*_•_^*p*^) for unknown dataset *A*.

For the same variables, there were identical distributions. As the mean concentrations of poultry serum proteins for the two datasets were different, the tendency of the poultry scores is observed. In this situation, (*Ax*_*i*_^*p*^ − mean(*Ax*_•_^*p*^))/S.D.(*Ax*_•_^*p*^) is the Z-score for the unknown dataset. However, the known set must have the same Z-score. Thus, (1) is generated.

Each variable in the known dataset and in the unknown dataset has two values. One is the transferred score, and the other is the transferred rank. The variable was chosen to find the similar objects in the unknown dataset. Theoretically, these two values should occur in the same object. However, they appear in the different objects. According to order transferred score *Ax* *t*_*pi*_^*p*^, the order transferred score and the transferred rank (Table 3(b)) generate one region.

**Figure 1:**
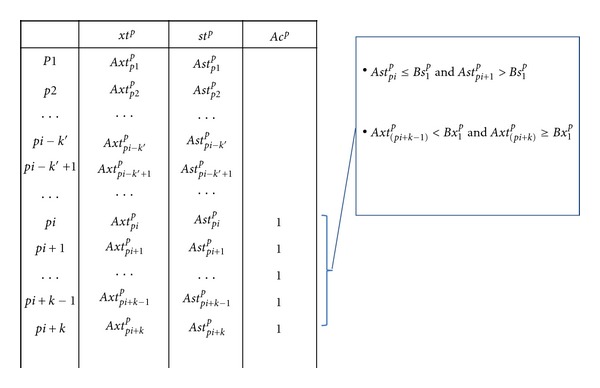
Find the first zone (*ej* = 1) from the order of the unknown dataset *A* at Case I. According to the score and rank, those two values of the objects were defined as the upper-bound and lower-bound of this region, respectively. A zone of “1” is used to define some of the objects that are between *pi* and *pi* + *k*. Therefore, we defined the zone of the object as {*Ac*_*pi*_^*p*^ = 1, *Ac*_*pi*+1_^*p*^ = 1,…, *Ac*_*pi*+*k*_^*p*^ = 1}.

**Figure 2:**
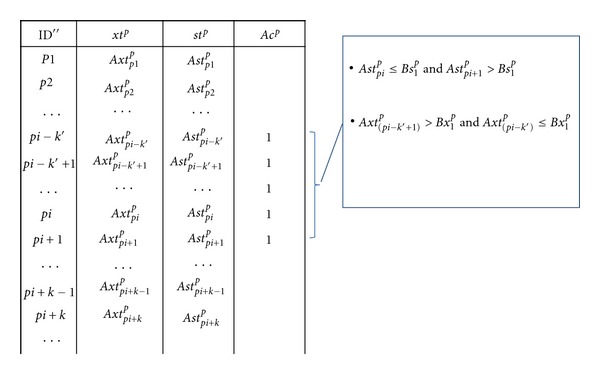
Find the first zone (*ej* = 1) from the order of the unknown dataset *A *at Case II. According to the score and rank, those two values of the objects were defined as the lower-bound and upper-bound of this region, respectively. A zone of “1” is used to define some of the objects that are between *pi* − *k*′ and *pi* + 1. Therefore, we defined the zone of the object as {*Ac*_*pi*−*k*′_^*p*^ = 1, *Ac*_*pi*−*k*′+1_^*p*^ = 1,…, *Ac*_*pi*+1_^*p*^ = 1}.

**Figure 3:**
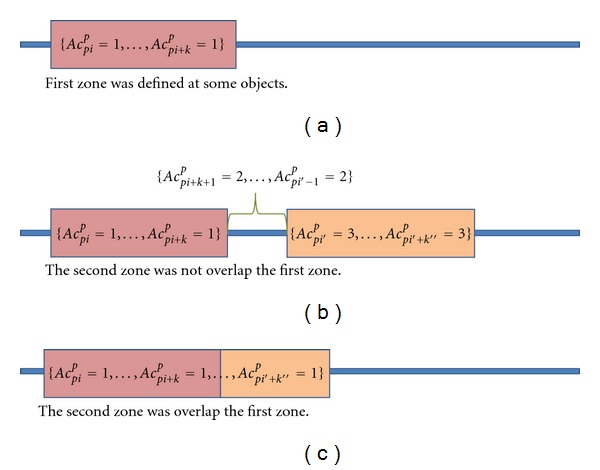
Generation of the zone.  3(a) shows a zone of “1” is used to define some of the objects. The second zone has two different cases.  3(b) shows the first case occurs where the second zone is not overlapping the first zone. The gap between these two regions is given a zone of “2”, where the two separated regions are assigned zones of “1” and “3”.  3(c) shows that the second case occurs when the second region overlaps the first zone.

Algorithm 2 at the step (2.1.1) and step (2.1.2) was described at Figures 1 and 2, respectively. Figures 1 and 2 show how to find the first zone (*ej* = 1) from the order of the unknown dataset A at Case I and Case II, respectively. The order transferred score and the transferred rank point to the different objects, and then these different objects become one region. According to the score and rank, those two values of the objects were defined as the upper-bound and lower-bound; or lower-bound and upper-bound of this region, respectively. Each object in the unknown dataset has one region number. If any region overlaps with another, these overlapping objects have same region number. For example, in the first zone, a zone of “1” is used to define some of the objects, as is shown in Figure 3(a). The second zone has two different cases. The first case occurs where the second zone is not overlapping the first zone as shown in Figure 3(b). The gap between these two regions is given a zone of “2”, where the two separated regions are assigned zones of “1” and “3”. The second case occurs when the second region overlaps the first zone as shown in Figure 3(c). In this circumstance, the two zones are combined to form a single region. The Algorithm then continues to find all the zone until *ej* = *cn*_*B*_.

We used the score and the rank of objects that are in the candidate dataset to obtain the zone. These zones use the rank order to obtain the order zones. Thus, the object in the candidate dataset has the *Bc*_*ej*_. Every object in the unknown dataset had its zone, *Ac*_*i*_ = {*Ac*_*i*_^1^, *Ac*_*i*_^2^,…, *Ac*_*i*_^*p*^,…, *Ac*_*i*_^*m*^}.

#### 2.2.3. Identifying Predicted Variables from the Zone

Each *p* is generated one number by Algorithm 1 or Algorithm 2, and every object includes *m* zones. The following algorithm used the zone to generate the predicted variables. PreZone chooses the same or more than number of validation candidates (*cn*_*B*_) as the number of predicted variables.

**Algorithm 3**

(1) Calculate the equation: *M*_*ej*,*i*_ = ∑_*p*=1_^*m*^|*Ac*_*i*_^*p*^ − *Bc*_*ej*_^*p*^|, where *ej* = 1,2,…, *cn*_*B*_ and *i* = 1,2,…, *n*_*A*_.

(2) Choose the predicted variables.

(2.1) If *M*_*ej*,*i*_ = 0 is calculated for a value of *ej*, then the object *i* is a predicted variable. If the total number of predicted variables is less than the number of validation candidates (*cn*_*B*_), the process will proceed to the next step (2.2).

(2.2) If *M*_*ej*,*i*_ ≠ 0 for any value of *ej* but *M*_*ej*,*i*_ = 1 is calculated for a value of *ej*, then the object *i* is the predicted variable. If the total number of predicted variables is less than *cn*_*B*_, the process will proceed to the next step (2.3).

(2.3) For a given value of *ej*, the variables are sorted by the value obtained by  *M*_*ej*,*i*_ in ascending order. From these a total of *cn*_*B*_ variables are selected. But there are same values obtained by *M*_*ej*,*i*_. This provides a set with a size of *W* elements. These elements are those variables with the smallest value of *M*_*ej*,*i*_. We can then identify what values of *i* exist in the set we have created. We then calculate the average value of *M*_*ej*,*i*_ for all values of *i* we have found in the set. If the *i* does not found in the set, the average value of *M*_*ej*,*i*_ is any one big number. We then search every set we create for a given value of *ej* and count how many times *i* appears. This provides us two values for each *i* that has been encountered. We then choose the smallest top third of the average *M*_*ej*,*i*_ and define that as the filter (F1) and then choose for a given value of *ej* the top third with the largest count of *i* and define this as a filter (F2). If for a given object both filters F1 and F2 are applied and afterward *M*_*ej*,*i*_ = 2 then the object *i* is the predicted variable. If the total number of predicted variables is less than *cn*_*B*_, the process will proceed to the next step (2.4).

(2.4) If *M*_*ej*,*i*_ = 2 and the object has either filter F1 or F2 applied, then object *i* is the predicted variable. If the total number of predicted variables is less than *cn*_*B*_, the process will proceed to the next step (2.5).

(2.5) We now choose the smallest top quarter of the average *M*_*ej*,*i*_ and define that as the filter (F3) and then choose for a given value of *ej* the top quarter with the largest count of *i* and define this as a filter (F4). If the object has the F3 and F4, then the object *i* is the predicted variable.

*M*_*ej*,*i*_  represents the difference between the unknown objects and the validation candidate dataset. When *M*_*ej*,*i*_ = 0, *i* is the predicted variable. If the number of the predicted variable is less than *cn*_*B*_, then we will use *M*_*ej*,*i*_ = 1 from step (2.2). According to *M*_*ej*,*i*_ for every *ej* we choose a set of *W* objects; however for each object chosen there are different values of*ej*. For each object and based on the sets we generate a series of four filters F1 to F4. The four filter conditions are defined in steps (2.3) to (2.5).

### 2.3. Materials

#### 2.3.1. Animal Housing and Measurement of Serum Protein Concentrations

The animal housing conditions and the methods for measuring serum protein markers were described by Liou et al. [17]. Briefly, three batches of TRFCCs, batch *A*(*n*_*A*_ = 76), batch *B* (*n*_*B*_ = 77), and batch *C* (*n*_*C*_ = 60) were included in this study.  Table 4 is the basic statistics analysis of serum protein concentrations for *A*, *B*, and *C* datasets. The average egg numbers for *A*, *B*, and *C* datasets were 94.57, 103.91, and 85.1, respectively. There were three datasets taken from three batches of birds. The birds in each batch were raised in different seasons and in different years. Total egg numbers were recorded individually and daily from 25 wks to 48 wks of age. Sera were collected from chickens at 14 and 24 weeks of age from batches *A* and *B*. In batch *C*, the sera were not collected at the same time as batch *A* and *B*. Sera for batch *C* were collected from chickens at 8, 14, and 22 weeks of age. The variables, measured at 8 wks and 14 wks of age, were the serum protein concentrations of apolipoprotein A-I, apo VLDL-II and the X protein; the concentration of vitellogenin was also included at other time stages. Previous reports showed that these proteins participate in egg formation [12, 18]. Vitellogenin and apo A-I are major components of yolk [19, 20]. Apo VLDL-II, a lipoprotein lipase inhibitor, plays an important role in VLDL transportation from the liver to the oocyte through the plasma [21]. X protein, an IGF-I-like protein, is associated with egg production [17]. Total egg number per chicken was served as the validation variable.

Tables 5, 6, and 7 are Pearson's correlation coefficient for *A*, *B*, and *C* datasets between all serum proteins, respectively. These tables show a low correlation between the number of eggs and all of the serum proteins. There is also a low correlation between each of the serum proteins, except in dataset *A* when the chickens were 24 weeks old.

#### 2.3.2. Ethics Statement

Full details of the study were approved by Animal Technology Institute Taiwan. All animal work had been conducted according to relevant national and international guidelines. Since the studied chickens were housing in private farm (Jin-Tai Livestock Co., LTD) in Taiwan between 2002 and 2003, the approval ID was not required during the study time period. The private farm is located at Yunlin in Southern Taiwan, and they gave approval for this study.

## 3. Results

There were three datasets (Table 4) taken from three batches of birds that were raised in the different seasons and years. There were 76 and 77 chickens in the *A* and *B* datasets, and the sampling time stages were 14 wks and 24 wks. The *C* dataset included data for 60 chickens; the sera were not collected at the same time as batches *A* and *B*. Sera for batch *C* were collected from chickens at 8, 14, and 22 weeks of age. The variables, measured at 8 wks and 14 wks of age, were the serum protein concentrations of apolipoprotein A-I, apo VLDL-II and the X protein; the concentration of vitellogenin was also included at other time stages. The average egg numbers for *A*,*B*, and *C* datasets were 94.57, 103.91, and 85.1, respectively.

There were two approaches used in this study. The first approach used the *B* dataset as a known set to select the low egg productivity, about *cn*_*B*_ = 9 (⌈77 × 0.1⌉ = 8 and the eighth egg order and ninth egg order are the same egg number), of birds in the *A* dataset. The second approach used union sets of the *A* and *B* datasets to select the low egg productivity of birds in the *C* dataset. Because the sampling time stages of the *A* and *B* datasets were different from that of the *C* dataset, we used *A* and *B* data at 14 wks to predict the *C* data at 8 wks and 14 wks. Because the intersection of sets *A* and *B* has the small predicted variables, there is another point of view that can be considered for the union of sets *A* and *B*. We also predicted the *C* data at 22 wks and 24 wks using 24 wks of data. In each approach, we used continuous selection methods. Continuous selection over time was defined as chickens were taken away at this time stage; then these chickens were not counted in the next time stage.

When we collect three datasets, we try to choose the low egg productivity chicken and to improve the egg productivity. We use the first-order multiple linear regression model (Table 8) to predict the egg productivity chickens. For example, if we want the data form set *A* at 14 wks to predict the data from set *B* at 14 weeks, we use the first equation and the *x*_1_, *x*_2_, *x*_3_  from dataset *B* to predict the egg umber. The egg productivity of the two datasets was generated, performed using the first-order multiple linear regression models, and the predicted expected variables were chosen by taking the same number of the PreZone predicted expected variables.

We use the first-order multiple linear regression models for predicting the low egg productivity in chickens, but this model cannot be used to improve egg productivity. As shown in Table 8, all the *P*-values are higher than 0.05 except for *A* dataset at 24 weeks. Therefore, we create a new PreZone method to predict egg productivity.  Table 9 shows the chosen values for batch *A* of TRFCCs calculated using the first-order multiple regression and PreZone method. Egg improvement as measured by both methods was higher in the mature stage (24 wks) than in the premature stage (14 wks) by chosen at continuous time stage. The PreZone could improve egg productivity by 2.8% for chickens that are 14 weeks old, and by 5% at 24 weeks old. The average egg numbers for *A* datasets were 97.172 and 99.235 at 14 weeks and 24 weeks by choosing low egg productivity. However, the regression method could only improve egg productivity by −0.2% and 3.6% at 14 weeks and 24 weeks, respectively. For chickens that are 24 weeks old, 68% of chickens that were chosen produced less than the average number of eggs using the prediction by zone method, while 61% of chickens produced less than the average number of eggs using the regression method. The average egg numbers for *A* datasets were 94.375 and 97.9375 at 14 weeks and 24 weeks by choosing low egg productivity.

Similar results are shown in Table 10. Obviously, the selection of *C* datasets by taking the union sets of *A* and *B* data could largely improve egg productivity using the PreZone on 8 wks and 14 wks of birds. The PreZone could improve egg productivity by 5.6% at 8 weeks old and by 8.6% at 14 weeks old. However, the regression method could only improve egg productivity by −3.5% and −3.4% at 8 weeks and 14 weeks, respectively. Selection of data *C* using union sets of *A* and *B* at three continuous time stages could improve egg productivity by 9.5%. Because the intersection of sets *A* and *B* has the small predicted variables, there is another point of view that can be considered for the union of sets *A* and *B*. The average egg numbers for *C* datasets were 89.9, 92.4, and 93.2 at 8 weeks, 14 weeks, and 22 weeks by choosing low egg productivity. In contrast, the selection of chickens using the regression method shows negative improvement of egg productivity during these stages. For chickens that are 22 weeks old, 68% of chosen chickens are producing less than the average number of eggs by the prediction by zone method. Using the regression method to improve egg productivity by −1.6%, 57% of chosen chickens, which are 22 weeks old, produced less than the average number of eggs. The average egg numbers for *C* datasets were 82.1, 82.2, and 83.7 at 8 weeks, 14 weeks and 22 weeks by choosing low egg productivity. These results imply that the accuracy of the selection of low egg productivity using the PreZone method is higher than the regression method used in the premature stage of birds.

## 4. Discussion

In the present study, we used a PreZone to improve the egg production in TRFCCs. Four serum proteins, vitellogenin, apolipoprotein A-I, X protein (an IGF-I-like protein), and apo VLDL-II, were used as chosen parameters for egg production in three batches of TRFCCs. The PreZone emphasises the individual variance among a population. Even though the zones associated with the low egg productivity of birds appeared irregularly in the two batches of birds, we could still find regularity of these zones in both populations based on score and rank transformations. Interestingly, at 8 and 14 wks of age, these serum proteins participate in body growth and development instead of egg formation. Moreover, no correlation was found between the levels of those serum proteins and egg numbers (Table 5 to Table 7). The regular tendency of those zones associated with low egg productivity in three batches of birds (8 and 14 wks) suggests that the individual variance might be programmed earlier, and a hen's potential for egg production seems to correspond to the levels of serum proteins. Although the expression of these proteins is regulated by upstream gene elements, gene polymorphisms that lead to differences in egg production and its association with the levels of serum proteins remain unclear.

The egg production rate has improved from 5% and 9.5% after two continuous (Table 9) and three (Table 10) stages (union set). Interestingly, the rate of egg production was also increased 5.6% or 8.6% or 9.5% by early-stage (8 to 22 wks) chosen. At this stage we only use three datasets, and the two of these datasets are used to predict the third dataset. If more datasets could be collected and combined then the accuracy could be improved. In Taiwan, TRFCCs enter the market around 14 wks old. The economic benefits will be evaluated in the future by zone method at those time stages.

In conclusion, in this paper we present the PreZone algorithm. The purpose of PreZone is to select chickens that produce low egg yield, based on serum protein levels as selection indices. Furthermore, if response and predictors have a low correlation, then PreZone provides an alternative prediction methodology.

## Figures and Tables

**Table:** (a) Unknown set *A*.

ID	*x* ^1^	*s* ^1^	*x* ^2^	*s* ^2^	⋯	*x* ^*p*^	*s* ^*p*^	⋯	*x* ^*m*^	*s* ^*m*^
*i* = 1	*Ax*_1_^1^	*As*_1_^1^	*Ax*_1_^2^	*As*_1_^2^		*Ax*_1_^*p*^	*As*_1_^*p*^		*Ax*_1_^*m*^	*As*_1_^*m*^
*i* = 2	*Ax*_2_^1^	*As*_2_^1^	*Ax*_2_^2^	*As*_2_^2^		*Ax*_2_^*p*^	*As*_2_^*p*^		*Ax*_2_^*m*^	*As*_2_^*m*^
*i* = *i*	*Ax*_*i*_^1^	*As*_*i*_^1^	*Ax*_*i*_^2^	*As*_*i*_^2^		*Ax*_*i*_^*p*^	*As*_*i*_^*p*^		*Ax*_*i*_^*m*^	*As*_*i*_^*m*^
⋮										
*i* = *n*_*A*_	*Ax*_*n*_*A*__^1^	*As*_*n*_*A*__^1^	*Ax*_*n*_*A*__^2^	*As*_*n*_*A*__^2^		*Ax*_*n*_*A*__^*p*^	*As*_*n*_*A*__^*p*^		*Ax*_*n*_*A*__^*m*^	*As*_*n*_*A*__^*m*^

**Table:** (b) known set *B*.

ID	*E*	*x* ^1^	*s* ^1^	*x* ^2^	*s* ^2^	⋯	*x* ^*p*^	*s* ^*p*^	⋯	*x* ^*m*^	*s* ^*m*^
*j* = 1	*E*_1_	*Bx*_1_^1^	*Bs*_1_^1^	*Bx*_1_^2^	*Bs*_1_^2^		*Bx*_1_^*p*^	*Bs*_1_^*p*^		*Bx*_1_^*m*^	*Bs*_1_^*m*^
*j* = 2	*E*_2_	*Bx*_2_^1^	*Bs*_2_^1^	*Bx*_2_^2^	*Bs*_2_^2^		*Bx*_2_^*p*^	*Bs*_2_^*p*^		*Bx*_2_^*m*^	*Bs*_2_^*m*^
*j* = *j*	*E*_*j*_	*Bx*_*i*_^1^	*Bs*_*i*_^1^	*Bx*_*i*_^2^	*Bs*_1_^2^		*Bx*_*i*_^*p*^	*Bs*_*i*_^*p*^		*Bx*_*i*_^*m*^	*Bs*_*i*_^*m*^
⋮											
*j* = *n*_*B*_	*E*_*n*_*B*__	*Bx*_*n*_*B*__^1^	*Bs*_*n*_*B*__^1^	*Bx*_*n*_*B*__^2^	*Bs*_*n*_*B*__^2^		*Bx*_*n*_*B*__^*p*^	*Bs*_*n*_*B*__^*p*^		*Bx*_*n*_*B*__^*m*^	*Bs*_*n*_*B*__^*m*^

**Table 2:** The validation candidate dataset. It shows the validation candidate dataset. The ID′ column is identity objects, but their order is dependent to the order of the validation variable, order *E*. We generated the candidate score variables, {*Bx*_*ej*_^1^,…, *Bx*_*ej*_^*p*^,…, *Bx*_*ej*_^*m*^}, and candidate rank variables {*Bs*_*ej*_^1^,…, *Bs*_*ej*_^*p*^,…, *Bs*_*ej*_^*m*^}.

ID′	Order *E *	*x* ^1^	*s* ^1^	*x* ^2^	*s* ^2^	⋯	*x* ^*p*^	*s* ^*p*^	⋯	*x* ^*m*^	*s* ^*m*^
*e1*	*E*_*e*1_	*Bx*_*e*1_^1^	*Bs*_*e*1_^1^	*Bx*_*e*1_^2^	*Bs*_*e*1_^2^		*Bx*_*e*1_^*p*^	*Bs*_*e*1_^*p*^		*Bx*_*e*1_^*m*^	*Bs*_*e*1_^*m*^
*e2*	*E*_*e*2_	*Bx*_*e*2_^1^	*Bs*_*e*2_^1^	*Bx*_*e*2_^2^	*Bs*_*e*2_^2^		*Bx*_*e*2_^*p*^	*Bs*_*e*2_^*p*^		*Bx*_*e*2_^*m*^	*Bs*_*e*2_^*m*^
*ej*	*E*_*ej*_	*Bx*_*ej*_^1^	*Bs*_*ej*_^1^	*Bx*_*ej*_^2^	*Bs*_*ej*_^2^		*Bx*_*ej*_^*p*^	*Bs*_*ej*_^*p*^		*Bx*_*ej*_^*m*^	*Bs*_*ej*_^*m*^
⋮											
*e_cnB_*	*E*_*e*_*cnB*__	*Bx*_*e*_*cnB*__^1^	*Bs*_*e*_*cnB*__^1^	*Bx*_*e*_*cnB*__^2^	*Bs*_*e*_*cnB*__^2^		*Bx*_*e*_*cnB*__^*p*^	*Bs*_*e*_*cnB*__^*p*^		*Bx*_*e*_*cnB*__^*m*^	*Bs*_*e*_*cnB*__^*m*^

**Table:** (a) The transferred unknown dataset *A*.

ID	*xt* ^*p*^	*st* ^*p*^
*i* = 1	*Ax* *t*_1_^*p*^	*As* *t*_1_^*p*^
*i* = 2	*Ax* *t*_2_^*p*^	*As* *t*_2_^*p*^
*i* = *i*	*Ax* *t*_*i*_^*p*^	*As* *t*_*i*_^*p*^
⋮		
*i* = *n*_*A*_	*Ax* *t*_*n*_*A*__^*p*^	*As* *t*_*n*_*A*__^*p*^

**Table:** (b) The order of the unknown dataset *A*.

ID′′	*xt* ^*p*^	*st* ^*p*^
*p1*	*Ax* *t*_*p*1_^*p*^	*As* *t*_*p*1_^*p*^
*p2*	*Ax* *t*_*p*2_^*p*^	*As* *t*_*p*2_^*p*^
*pi*	*Ax* *t*_*pi*_^*p*^	*As* *t*_*pi*_^*p*^
⋮		
*pn*_*A*_	*Ax* *t*_*pn*_*A*__^*p*^	*As* *t*_*pn*_*A*__^*p*^

**Table 4:** A basic statistical analysis for the serum protein concentrations for the *A*, *B*, and *C* dataset.

Dataset	Week old	Number of objects	Number of missing objects	Apo A-I		X protein		Apo VLDL-II		Vitellogenin	
				$\overset{®}{X}$	*σ*	$\overset{®}{X}$	*σ*	$\overset{®}{X}$	*σ*	$\overset{®}{X}$	*σ*
*A*	14	71	5	1.726	0.347	0.245	0.203	0.043	0.080	—	—
	24	76	0	2.710	1.684	0.720	0.470	0.200	0.190	0.813	0.906

*B*	14	76	1	2.641	0.732	0.594	0.293	0.035	0.049	—	—
	24	77	0	2.219	1.083	1.292	0.410	0.374	0.300	1.036	0.786

*C*	8	60	0	2.752	0.894	0.169	0.087	0.026	0.033	—	—
	14	60	0	2.156	0.311	0.416	0.216	0.024	0.031	—	—
	22	60	0	2.631	0.854	0.871	0.490	0.316	0.342	0.494	0.482

$\overset{®}{X}$: mean; *σ*: standard deviation.

**Table 5:** The Pearson's correlation coefficient of *A* dataset is between serum protein concentrations apolipoprotein A-I, apo VLDL-II, X protein and Vitellogenin.

		A 14 weeks			A 24 weeks			
		Apo A-I	VLDL-II	*X *	Apo A-I	VLDL-II	*X *	Vite
*A* 14 weeks	VLDL-II	−0.101						
	*X *	−0.139	0.216					
	egg	0.156	−0.154	−0.162				

*A* 24 weeks	VLDL-II				−0.481			
	*X *				−0.551	0.545		
	vite				−0.520	0.732	0.604	
	egg				0.198	0.195	0.126	0.240

Apo A-I: apolipoprotein A-I; VLDL-II: apo VLDL-II; X: X protein; vite: vitellogenin.

**Table 6:** The Pearson's correlation coefficient of *B* dataset is between serum protein concentrations apolipoprotein A-I, apo VLDL-II, X protein and Vitellogenin.

		B 14 weeks			B 24 weeks			
		Apo A-I	VLDL-II	*X *	Apo A-I	VLDL-II	*X *	Vite
*B* 14 weeks	VLDL-II	−0.040						
	*X *	−0.248	0.207					
	egg	0.198	0.135	0.04				

*B* 24 weeks	VLDL-II				−0.268			
	*X *				0.103	0.247		
	vite				0.242	−0.282	0.088	
	egg				0.071	0.145	0.053	0.029

Apo A-I: apolipoprotein A-I; VLDL-II: apo VLDL-II; *X*: *X *protein; vite: vitellogenin.

**Table 7:** The Pearson's correlation coefficient of *C* dataset is between serum protein concentrations apolipoprotein A-I, apo VLDL-II, X protein and Vitellogenin.

		C 8 weeks			C 14 weeks			C 22 weeks			
		Apo A-I	VLDL-II	*X *	Apo A-I	VLDL-II	*X *	Apo A-I	VLDL-II	*X *	vite
*C* 8 weeks	VLDL-II	0.231									
	*X *	0.089	0.100								
	egg	−0.206	−0.167	−0.011							

*C* 14 weeks	VLDL-II				−0.040						
	*X *				−0.248	0.207					
	egg				0.198	0.135	0.040				

*C* 22 weeks	VLDL-II							−0.268			
	*X *							0.103	0.247		
	vite							0.242	−0.282	0.088	
	egg							0.071	0.145	0.053	0.029

Apo A-I: apolipoprotein A-I; VLDL-II: apo VLDL-II;* X*: *X *protein; vite: vitellogenin.

**Table 8:** First-order multiple linear regression model.

Dataset	Week old	Regression equation^a^	*P*-value
A	14	egg = 87.1 + 6.69*x*_1_ − 25.2*x*_2_ − 15.1*x*_3_	0.195
A	24	egg = 66 + 5.87*x*_1_ + 13.4*x*_2_ + 5.95*x*_3_ + 6.97*x*_4_	0.001
B	14	egg = 87.2 + 4.82*x*_1_ + 54*x*_2_ + 4.98*x*_3_	0.293
B	24	egg = 93.4 + 2.10*x*_1_ + 13.4*x*_2_ − 0.58*x*_3_ + 1.5*x*_4_	0.603

^a^the *x*_1_, *x*_2_, *x*_3_, and *x*_4_ are serum protein concentrations. *x*_1_ is the apolipoprotein A-I, *x*_2_ is the VLDL-II, *x*_3_ is the *X* protein. *X*_4_ is the vitellogenin.

**Table 9:** Selection of low egg productivity in batch *A* of birds by regression and PreZone method.

Weeks old	Regression				PreZone			
	Average egg no.	No. of selected birds	Number of selected birds under average egg no. (percentage)	Egg improvement^b^	Average egg no.	No. of selected birds	No. of selected birds under average egg no. (percentage)	Egg improvement
Approach^a^								
14	94.375	12	3 (3/12 = 25%)	−0.2%	97.172	12	9 (9/12 = 75%)	2.8%
24	97.9375	28	17 (17/28 = 61%)	3.6%	99.235	25	17 (17/25 = 68%)	5%

^a^Selection approach at continuous two-time stages.

^b^Average egg number after birds selected divided by original average egg number (94.57). For example, (94.375 − 94.57)/94.57 = −0.2% and (97.172 − 94.57)/94.57 = 2.8%.

**Table 10:** Selection of low egg productivity in batch *C* of birds using union set of batches *A* and *B*.

Weeks old	Regression				PreZone			
	Average egg no.	No. of selected birds	No. of selected birds under average egg no.	Egg improvement^b^	Average egg no.	No. of selected birds	No. of selected birds under average egg no.	Egg improvement
Union set								
Approach^a^								
8	82.1	17	9 (9/17 = 53%)	−3.5%	89.9	19	15 (15/19 = 79%)	5.6%
14	82.2	29	17 (17/29 = 59%)	−3.4%	92.4	31	22 (22/31 = 71%)	8.6%
22	83.7	37	21 (21/37 = 57%)	−1.6%	93.2	37	25 (25/37 = 68%)	9.5%

^a^Selection approach at continuous three time stages.

^b^Average egg number after birds selected divided by original average egg number (85.1). For example, (82.1 − 85.1)/85.1 = −3.5% and (89.9 − 85.1)/85.1 = 5.6%.
